# BRIDGING HEALTH EQUITY GAPS: CULTURAL TAILORING AND COMMUNITY-BASED APPROACHES WITH DIVERSE OLDER ADULTS

**DOI:** 10.1093/geroni/igae098.0871

**Published:** 2024-12-31

**Authors:** Ryan Mace, Ana-Maria Vranceanu, Kathi Heffner

**Affiliations:** Chair; Co-Chair; Discussant

## Abstract

Chronic conditions and related stressors disproportionately affect underserved older adult communities, increasing their risk for adverse health outcomes and emphasizing the need for community engagement and culturally tailored interventions. Consistent with the GSA 2024 conference theme, this symposium will spotlight multidisciplinary initiatives that utilize community engagement and cultural tailoring as “Fortitude Factors” to enhance health equity, quality of life, and well-being in diverse older populations. Presenter 1 will discuss a pilot effectiveness trial of “GetActive,” a culturally adapted program for chronic pain management in older adults delivered in Spanish and English, showing improved pain management and quality of life. Presenter 2 will present findings from an ongoing community-engaged study providing transportation support through Uber to address inequities faced by home care workers for those with cognitive impairments, highlighting structural challenges. Presenter 3 will report on the effectiveness of the “PEERS” program for minority and low-income older adults with depression, underscoring the value of social support, particularly during the COVID-19 pandemic. Presenter 4 will report on a qualitative study that identifies and proposes strategies to mitigate socio-ecological barriers within a dementia prevention program “My Healthy Brain”, emphasizing the need for a multi-level approach to enhance equity in dementia clinical trials. The Discussant will situate these studies within the broader field of gerontology, highlighting their potential to address disparities and enhance behavioral interventions for diverse aging populations.

